# The PJ Nicholoff Steroid Protocol for Duchenne and Becker Muscular Dystrophy and Adrenal Suppression

**DOI:** 10.1371/currents.md.d18deef7dac96ed135e0dc8739917b6e

**Published:** 2017-06-27

**Authors:** Kathi Kinnett, Garey Noritz

**Affiliations:** Parent Project Muscular Dystrophy, Hackensack, New Jersey, USA; Complex Care, Nationwide Children's Hospital, Columbus, OH, USA

## Abstract

Duchenne muscular dystrophy (DMD or Duchenne) is a progressive, life-limiting muscle-wasting disease that requires comprehensive, multidisciplinary care. This care, at minimum, should include neuromuscular, respiratory, cardiac, orthopedic, endocrine and rehabilitative interventions that address both the primary and secondary manifestations of the disease. The care needs of patients evolve over the cdourse of the disease and as they transition from childhood into young adulthood. In the past two decades, life expectancy has increased significantly by the use of corticosteroids and enhanced clinical management. Nevertheless, each year, patients with Duchenne muscular dystrophy are admitted to emergency departments and intensive care units where medical expertise thrives, but where expertise in rare diseases, such as Duchenne, may not. Emergency care for patients with Duchenne can be as complex as the disease process itself. While any illness or injury may occur in a person with Duchenne, some acute scenarios are much more common in the context of the disease. Making decisions about the clinical care of a person with Duchenne who presents with an acute illness can be quite difficult — in part, because of the extensive use of corticosteroids, which can lead to adrenal suppression. The life of a person with Duchenne needing emergency care may therefore depend upon the ability of the clinician on duty in the emergency department to recognize and mitigate adrenal suppression resulting from corticosteroid dependence. With this in mind, and drawing from expertise and experience with other steroid-dependent diseases, the ‘PJ Nicholoff Steroid Protocol’ was developed. The purpose of this protocol is to provide clinicians information regarding the safe management of corticosteroid during emergency situations in patients who may have accompanying adrenal suppression. The protocol explains how to recognize the signs and symptoms of acute adrenal crisis, how to prevent it with supplemental stress doses of corticosteroids, and how to taper doses after emergency care in order to prevent corticosteroid withdrawal.

## Introduction

Duchenne Muscular Dystrophy (DMD) is the most common and severe muscle disease presenting in childhood. It is caused by mutations in the dystrophin gene, located on the X chromosome, which causes a complete absence of dystrophin protein in muscle. Becker Muscular Dystrophy (BMD) is caused by partial absence of dystrophin; this disease is less severe and less common. As an X-linked disease, both diseases almost always affect males, though some females may be affected as well. Duchenne and Becker have a prevalence of 1 in 5000 males, in about a 2:1 ratio.^1^^,^^2^

People with Duchenne Muscular Dystrophy have progressive muscle weakness, which generally begins proximally and spreads to the legs, arms and other muscles. It is now known from a newborn screening study that the onset of disease is at birth, and probably *in utero*.^3^ Boys with Duchenne are born with serum creatine kinase (CK) values over 2000 U·L-1. The diagnosis is typically made between 3 and 5 years of age. Progressive muscle weakness leads to the loss of ambulation at 10 to 12 years old without treatment, though ambulation can be extended by one or two years through the use of corticosteroids. There is progressive respiratory muscle weakness, which leads to hypoventilation, respiratory insufficiency, and respiratory failure in adulthood. There is also a progressive cardiomyopathy leading to cardiac insufficiency. Prior to the use of corticosteroids, assisted ventilation and other interventions, the median life expectancy was age 19; however, today most deaths are due to heart disease and heart failure, and typically occur in the mid-20s, though survival into the 30s is no longer uncommon. 4^,^^5^

In the late 1980’s, it was established that corticosteroids could improve and prolong ambulation and slow the rate of decline in both Duchenne and Becker.^6^ The use of corticosteroids has also reduced the incidence and severity of scoliosis. However, chronic use of corticosteroids is associated with significant side effects that require monitoring and treatment.

International consensus recommendations on the comprehensive care for people living with Duchenne were published in Lancet Neurology in 2010. 5^,^^7^ A summary listing the key elements of Duchenne care was published in this journal in 2015.^8^

Given the multi-systemic nature of the disease, there should generally be an interdisciplinary team of care providers with whom to consult, including, at minimum, a coordinator, neurologist, doctor of physical medicine and rehabilitation, pulmonologist, cardiologist, social worker, dietician and primary care doctor. However, one issue in the management of Duchenne is that due to increased life expectancy, patients at some point need to transition out of pediatric care and on to adult care teams. Although the transition into adult care should be a process, clinicians in the emergency department should be aware that it is sometimes delayed or incomplete — and may in fact be triggered by the very acute illness or clinical event that brings them to the emergency room (ER).

While any illness or trauma may occur in a person with Duchenne or Becker, certain clinical scenarios are more common. These include exacerbations of the underlying respiratory or cardiac disease; kidney stones; and fractures as a result of low bone mineral density. Patients with Duchenne also seem to be prone to a particularly devastating consequence of fracture, fat embolism syndrome.^9^^,^^10^ Each medical crisis needs to be evaluated and responded to in the context of the expected complications of the disease or medications used for disease management — particularly the chronic use of corticosteroids, which can lead to suppression of the hypothalamus-pituitary-adrenal axis (HPA).

One danger is that, as pressing medical emergencies emerge, issues of daily medical care (including medications) may be pushed aside. However, the consequences of suddenly discontinuing or not appropriately dosing corticosteroids, particularly during a crisis can be quite severe, as the patient may have little to no ability to produce cortisol in response to stress. An additional issue for emergency departments is that many patients with Duchenne or Becker use Deflazacort, a corticosteroid that was recently approved in the United States but may be unfamiliar to doctors.

A recent case in point involved Phillip James “PJ” Nicholoff, a vibrant, 31-year-old man living with Duchenne muscular dystrophy. PJ had been treated with daily corticosteroids since the age of 6. He endured several pathologic fractures, likely a result of his steroid treatment and his non-ambulatory status. In November 2013, on a trip to Florida, he fractured his humerus and hip, and was transported by plane to a hospital closer to his home in the north. PJ had orthopedic surgery to manage both of these fractures. Following the surgery, however, he developed respiratory distress, tachycardia, hypotension, and later died. A review of the medical record suggested that he had not received appropriate stress-doses of steroids during his hospitalization. While PJ’s death may have been attributed to many causes, inadequate corticosteroid doses may have been a contributing factor.. Consequently, experts in the field held extensive discussions in order to develop a protocol on steroid dosing during an acute illness or other emergency.

The protocol addresses several issues:

- How to define HPA suppression in a patient using corticosteroids
- Appropriate corticosteroid stress doses for minor, moderate, and major stressors
- Recommendations for corticosteroid withdrawal
- How to test the HPA axis for continued suppression
- Symptoms of acute adrenal crisis
- Tests that can help diagnose adrenal crisis
- Corticosteroid conversions/equivalent doses

**The PJ Nicholoff Steroid Protocol**

**1. Background/Assessment**

The normal basal secretion of cortisol from the adrenal gland is approximately 5-7 mg/m2/day in children or 8 -10 mg/day for adults.^11^^,^^12^ This amount increases during minor illnesses or surgery to approximately 50 mg/day (5x normal physiologic secretion), and typically return to baseline in 24 hours. Severe illness, trauma, or major surgical procedures cause increased cortisol production to about 75-150 mg/day (10x normal physiologic secretion), which return to baseline in about 5 days.^13^^,^^14^

Long-term administration of corticosteroids (primarily prednisone, prednisolone or deflazacort) may lead to suppression of the hypothalamic-pituitary-adrenal (HPA) axis, such that the adrenal glands no longer produce endogenous cortisol. Rapid reduction or abrupt withdrawal of corticosteroid therapy can cause secondary adrenal insufficiency and steroid withdrawal or deprivation syndrome, which may progress to adrenal crisis. Adrenal crisis is characterized by hypotension and hypovolemic shock, which may be life threatening.^15^ Recovery from suppression of the HPA axis after discontinuing corticosteroids can be prolonged (possibly 6 to 12 months) and may vary based on doses, dosing schedules and duration of corticosteroid therapy.^14^ Since there is a great deal of individual variability, it is not possible to predict with confidence which patients will be affected by adrenal suppression. Current practice is to administer supplemental (stress) doses of corticosteroids to patients with possible suppression of the HPA axis in the perioperative period and during acute illness to prevent acute adrenal insufficiency, or adrenal crisis.

**2. Defining HPA Suppressed Patients:**

Recommendations differ slightly in defining a suppressed patient, but general guidelines are as shown in table 1.^16^ These recommendations are primarily based on expert opinion and practice, as little data in this area have been published, but are based on the degree of medical/surgical stress and the likelihood of adrenal suppression.^16^ Consultation with an endocrinologist is recommended for questions or concerns.

**Figure:**
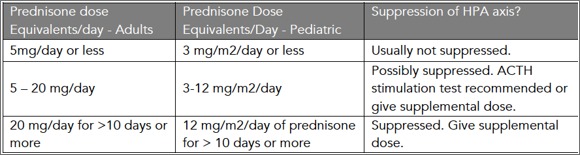
**Table 1**: Defining Adrenal Suppression.^16^ Note: doses for prednisone and prednisolone are equivalent; conversion is required for patients taking deflzacort (5 mg prednisone or prednisolone = 6 mg deflazacort)

Patients receiving disease appropriate corticosteroid doses (at least 10 times above the physiologic cortisol dose) generally do not need stress doses if usual daily dose is continued. Patients who are on maintenance physiologic dose of hydrocortisone for primary disease of the HPA axis do require supplemental therapy.^17^ Recommendations for supplemental doses are generally divided by the severity of the stress the patient may experience (medical or surgical).

**3. Corticosteroid Stress Doses for Patients Using Chronic Daily Corticosteroids**

**Figure:**
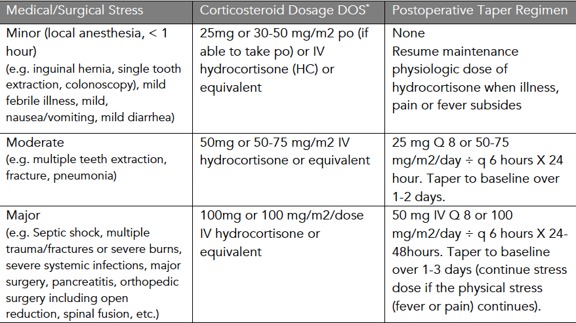
**Table 2**: Corticosteroid Stress Dosing

For patients using a high dose, twice-weekly corticosteroid^*^:

- If patients are unable to take their usual corticosteroids by mouth due to nausea, vomiting, or npo status, they should take stress doses intravenously as indicated above.
- During a moderate or major medical or surgical stressor, a cortisol level should be drawn prior to stress dosing.

**4. Recommendation for withdrawal of Corticosteroid Therapy:**

- It is recommended that patients electing to discontinue the use of corticosteroids do so under the guidance of a neuromuscular provider and/or endocrinologist.

Weaning from corticosteroids, and reactivating the adrenal glands, may take several months to achieve. A recommendation for tapering chronic corticosteroids (generally managed in an outpatient setting) is as follows:

- First, starting on a Monday, giving 20-25% lower corticosteroid dose for 2 weeks (or longer)
- Next, if multiple daily doses are taken, start first to reduce to a single morning dose
  - Cut the dose 20-25% again for 2 weeks (or longer); continues this schedule
  - Continue until dose is near physiologic dose (3mg/m2/day of prednisone or 3.6mg/m2/day of Deflazacort)
- When near physiologic dose, substitute corticosteroids with short acting form of corticosteroid or hydrocortisone (12 mg/m2/day of hydrocortisone)
- This will also enable the patient to have a supply of hydrocortisone to be used for stress doses if needed in times of stress after coming off steroids
  - Continue to taper off by 20-25% each week (or longer)
  - Give every other day for 2 weeks (or longer)
  - Stop
  - *Watch very carefully for signs of adrenal crisis (see below)*
- Alert patients and parents to signs/symptoms of adrenal crisis
- If patients have symptoms of adrenal insufficiency during the taper, the patient should return to the previous steroid dose, which should be maintained longer

*If the patient has a serious illness/injury during the taper, they may need a “stress dose” of corticosteroids:*

- Encourage parents to continue to report any serious events until 1 year post-taper
  - The stress doses of hydrocortisone is 30-50 mg/m2/day, or higher, for major stress (see Table 2)
  - Patients need to go to the emergency room if they are having signs or symptoms of adrenal crisis. Serum electrolytes with blood glucose and cortisol level should be obtained.
  - While it is appropriate to assess for acute adrenal crisis, this assessment should never delay treatment with a stress dose of hydrocortisone.
  - Patients should see an endocrinologist for evaluation of HPA axis during the process of corticosteroid therapy withdrawal.

Testing the HPA axis:^18^

- After reaching half the physiological dose (5-6 mg/m2/day of hydrocortisone or 1-1.5 mg/m2/day of prednisone), monthly morning serum cortisol and ACTH should be assayed (may do less frequently), until they reach normal levels.
- When baseline monthly morning serum ACTH and cortisol are normal, discontinue the corticosteroid and carry out the rapid ACTH stimulation test (may also be called cosyntropin, tetracosactide or Synacthen tests) monthly until post-stimulation cortisol response is normal (post-stimulus level > 20 mcg/dL). When this point is reached, it can be considered that the HPA axis has recovered.
- It may be appropriate to recheck the rapid ACTH stimulation test after 1 year, to establish continued full recovery of the HPA axis.

## Modification of above protocol:

- Omit monthly AM cortisol and ACTH and perform an ACTH stimulation test in 3 months after discontinuation of corticosteroids
- During this time (3 months before getting ACTH stimulation test), patients will need to take stress dose at the time of stress
- If ACTH stimulation test result is abnormal (peak cortisol <20), patients will need to continue taking stress doses of hydrocortisone at the time of stress. (Patients should have a repeat ACTH stimulation test again in 1-2 months later and families would need to have teaching on this with an endocrine nurse.)

Alternatively, when laboratory tests cannot be carried out:

- Patients who have used corticosteroids for prolonged periods can be considered as having suppression of the HPA axis up to 1 year after discontinuation of corticosteroid therapy and therefore need hydrocortisone stress dose coverage during the time of stress.

Risk factors for adrenal crisis include:^19^

- Dehydration
- Infection and other physical illness
- Injury to the adrenal or pituitary gland
- Missing usual doses of corticosteroids
- Surgery
- Trauma

Symptoms of adrenal crisis can include any of the following:^15^

- Abdominal pain
- Hypovolemic shock
- Confusion or coma
- Dehydration
- Dizziness or light-headedness
- Fatigue
- Flank pain
- Headache
- High fever
- Loss of appetite
- Loss of consciousness
- Severe hypotension
- Nausea
- Profound weakness
- Tachycardia
- Tachypnea
- Slow, sluggish movement
- Unusual and excessive sweating on face or palms
- Vomiting

Exams and Tests

While it is appropriate to assess for acute adrenal crisis, this assessment should never delay treatment with a stress does of hydrocortisone.

Tests that may be ordered to help diagnose acute adrenal crisis include:

- ACTH (cosyntropin) stimulation test
- Cortisol level
- Blood glucose
- Serum potassium
- Serum sodium
- Serum pH

**Figure:**
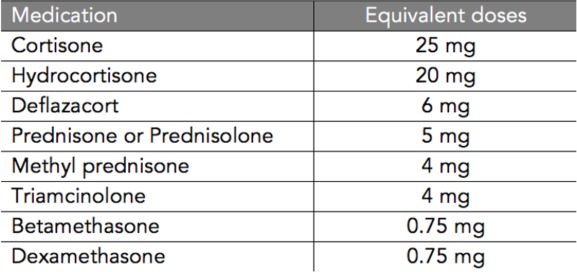
**Table 3**: Corticosteroid Conversion Table

## Conclusion

This protocol, on how to manage patients with corticosteroid dependence, particularly during periods of stress and how to recognize and prevent an acute adrenal crisis, was named in honor of the late Philip James “PJ” Nicholoff, for his contribution to the global Duchenne community. Despite the tragic loss of PJ’s life, the “PJ Nicholoff Steroid Protocol” is a positive result that will impact the lives of people living with Duchenne and using corticosteroids around the world. We thank PJ and his family for encouraging the development of this resource.

**A pdf of the PJ Nicholoff Protocol can be downloaded at: http://www.parentprojectmd.org/site/DocServer/PJ_Nicholoff_Steroid_Protocol.pdf?docID=15843Acknowledgements for the development of the PJ Nicholoff Steroid Protocol**

**** *In honor of the late Philip James “PJ” Nicholoff, for his contribution to the global Duchenne community.*

- Vincent’s Hospital, Indianapolis, IN
- Philip Zeitler, Children’s Hospital Colorado, Aurora, CO
- Sasigarn Bowden, Nationwide Children’s Hospital, Columbus, OH
- Doug Biggar, Holland Bloorview Kids Rehab, Toronto, ON
- Jerry R. Mendell, Nationwide Children’s Hospital, Columbus, OH
- Anne M. Connolly, St. Louis Children’s Hospital, St. Louis, MO

**Supporting Organizations**

**Parent Project Muscular Dystrophy (PPMD)**

Parent Project Muscular Dystrophy (PPMD, www.parentprojectmd.org) is the largest nonprofit organization in the United States focused entirely on Duchenne. Started in 1994 by Pat Furlong, PPMD takes a comprehensive approach in the fight against Duchenne—funding research, raising awareness, promoting advocacy, connecting the community, and broadening treatment options. PPMD’s care objectives are to identify gaps in care for people with Duchenne and work toward solutions, and to work with clinicians and other health care professionals across the globe to ensure all Duchenne patients have access to optimal care.

## Competing Interests Statement

The Authors have declared that no competing interests exist.

## Corresponding Author

Kathi Kinnett (kathi@parentprojectmd.org)
